# Extemporaneous Preparation and Effectiveness of Low-Dose Naltrexone for the Treatment of Uremic Pruritus: A Literature Review and Case Report

**DOI:** 10.3390/pharmacy13060160

**Published:** 2025-11-01

**Authors:** Dhakrit Rungkitwattanakul, Michelle Brooks, Simeon Adesina, Sanaa Belrhiti, Weerachai Chaijamorn, Taniya Charoensareerat, Uzoamaka Nwaogwugwu, Constance Mere

**Affiliations:** 1 Department of Clinical and Administrative Pharmacy Science, Howard University College of Pharmacy, Washington, DC 20059, USA; 2 Department of Pharmacy, Howard University Hospital, Washington, DC 20060, USA; 3 Department of Pharmaceutical Sciences, Howard University College of Pharmacy, Washington, DC 20059, USA; 4 Department of Pharmacy Practice, Faculty of Pharmaceutical Sciences, Chulalongkorn University, Bangkok 10330, Thailand; 5 Faculty of Pharmacy, Siam University, Bangkok 10160, Thailand; 6 Division of Nephrology, Department of Medicine, Howard University College of Medicine, Washington, DC 20059, USA

**Keywords:** uremic pruritus, naltrexone, low dose, hemodialysis

## Abstract

Background: Uremic pruritus is one of the most debilitating complications among patients with end-stage kidney disease (ESKD) receiving hemodialysis. For patients who are refractory to traditional therapies (topical analgesics, antihistamines, or gabapentinoids), the use of low-dose naltrexone can be an option where difelikefalin is not available. Case report: In our case report, we present a case of a female patient who developed intractable uremic pruritus despite the adequate trials of traditional therapies. The patient was initiated with low-dose naltrexone of 5 mg daily. Uremic symptoms improved within 3 days of naltrexone initiation. The side effects were tolerated. Conclusion: Low-dose naltrexone provided symptomatic improvement in individuals with severe uremic pruritus when difelikefalin was inaccessible. While limited to a single case, this report highlights the potential role of naltrexone and underscores the need for further research to establish its safety and efficacy.

## 1. Background

Uremic pruritus is one of the most debilitating complications among end-stage kidney disease (ESKD) patients receiving hemodialysis (HD) [1]. It is a distressing disorder that affects multiple aspects of quality of life, including sleep, mood, appetite, volume status, and social relationships [1,2]. Unfortunately, both pathophysiology of uremic pruritus and its treatment remain poorly understood.

Risk factors for uremic pruritus among hemodialysis patients include older age, female sex, inadequate dialysis, hyperparathyroidism, elevated [calcium × phosphate] product, and the elevation of serum magnesium and aluminum [3]. The pathophysiology behind uremic pruritus is thought to be explained by complex interactions between itch sensory pathways including immunologic and opioidergic systems [4,5]. The opioid hypothesis involves imbalances in the expression of mu and kappa opioid receptors, causing pruritus. This phenomenon is driven by an increase in the amount of mu (µ) receptors and a reduction in kappa (Ƙ) receptors, both of which are found in hemodialysis patients [4,5]. Therefore, uremic pruritus is alleviated by the blockade of mu receptors or the activation of Ƙ receptors. Immune dysregulation plays a critical role in uremic pruritus. Compared with non-pruritic patients, C-reactive protein and other inflammatory mediators including interleukin (IL)-2 and IL-6 are higher among pruritus patients [6,7].

The treatment of uremic pruritus is currently limited. There was no medication approved by United States (US) Food and Drug Administration (FDA) until difelikefalin was approved in 2021 [8]. Conventional therapies addressing pathophysiology, such as emollient treatment, antihistamine use, gabapentinoid treatment, phototherapy, and optimizing dialysis, have been suggested. However, the data are either from observational studies or small randomized controlled trials. Difelikefalin is the only FDA-approved medication for uremic pruritus in ESKD patients on HD [8,9]. Difelikefalin acts as a selective, peripherally acting kappa receptor agonist and was shown to significantly reduce pruritus severity [8,9]. Unfortunately, the medication is approved to be used in an outpatient setting and is only available as an outpatient treatment that can be given at the HD center. Therefore, patients who are admitted to the hospital have limited access to this medication. Over the past decade, accumulating evidence recommends the use of naltrexone as a therapeutic option for the treatment of dermatologic diseases [10]. Naltrexone is not FDA-approved for the treatment of any primary skin disease. However, several models showed that naltrexone modulates and sensitizes proinflammatory cytokines, thereby reducing the sensitivity and pruritus severity [11]. At low doses, naltrexone has shown benefit in certain chronic dermatologic conditions (e.g., prurigo nodularis, psoriasis), supporting the idea that it can modulate inflammatory-driven itch [10,11]. Naltrexone was originally approved for the treatment of alcohol and opioid use disorders at the dose of 50 mg daily. At a 50 mg/day dose, naltrexone blocks the euphoric effects of opioid for 24 h per dose. However, at the lower dose of 5 mg, naltrexone partially antagonizes μ, κ, and δ opioid receptors for 2 to 6 h per dose [11]. This primary effect of low-dose naltrexone offers a promising treatment approach to alleviate the severity of uremic pruritus. In the USA, naltrexone is commercially available as a 50 mg plain tablet [11]. For patients requiring lower doses, it often requires compounding which unfortunately comes with a significant price increase at the compounding pharmacies and is not covered by many insurance plans. By combining this literature review and a case report, we aim to review the evidence of naltrexone effectiveness for the treatment of uremic pruritus and present the case of a patient who had uremic pruritus that was refractory to conventional treatments and was responsive to low-dose naltrexone. We also aim to present suggestions for the inexpensive extemporaneous preparation of low-dose naltrexone based on our case report and observations.

## 2. Case Report

The case is a 68-year-old woman who presented to the hospital with generalized body itchiness, which started while she received hemodialysis. She has a past medical history of anthracycline-induced nonischemic cardiomyopathy, heart failure with reduced ejection fraction (ejection fraction of 30–35%, measured at this admission), breast cancer (in remission), anemia, thrombocytopenia, and chronic hypotension on midodrine. She has no history of hepatitis or human immunodeficiency virus infection (HIV). She denies shortness of breath, chest pain, fever, cough, leg swelling, nausea, and vomiting. She has no history of drug allergies. Her home medications include allopurinol 100 mg daily, apixaban 5 mg two times daily, amiodarone 400 mg two times daily, calcium acetate 1334 mg three times daily with meals, ferric citrate 630 mg three times daily with meals, hydroxyzine 50 mg three times daily, and rosuvastatin 10 mg daily.

### 2.1. Pruritus History

The patient has a history of ESKD, receiving conventional hemodialysis three times a week. With regard to her itchiness, she has been experiencing itching for a duration of a 2 years. This previously was localized to her legs and back, but recently, in the past few days prior to this admission, it spread to bilateral feet. She reported that dialysis worsened her itch, but she itched constantly all day. The patient rated itch at a 10/10 (with 10 being worse than she can handle). Her itch improves in environments like inside the car, and when water is applied. She denied worsening or improvement with heat, exercise or sweating. She denied pain, numbness or tingling. She also denied recreational drug use or travel.

Gabapentin 100 mg PO TID was started by a dermatologist from outside the hospital setting and minimally improved symptoms. The hydroxyzine and diphenhydramine started during the inpatient care were not effective in controlling her itch, per the patient’s report.

### 2.2. Diagnosis, Pertinent Vital Signs, Laboratory Results, Medications, and Visual Analog Scale (VAS) on Itchiness Level

The patient was initially admitted to the general medicine service (hospital medicine). The primary team consulted both the nephrology and dermatology teams for the further management of suspected uremic pruritus. Upon admission, the patient initiated hemodialysis with a similar prescription from her chronic outpatient treatments, as recommended by the nephrology team (comparable blood flow and dialysate flow rates). Ultrafiltration rates were set as tolerated by the patient and patient’s clinical status. The patient’s pruritus was assessed by dermatology team by both physical examination and history taking daily. The dermatology team decided to postpone skin biopsy and opted to manage the patient conservatively by medication therapy first. As shown in Table 1, the patient has an appropriate metabolic panel throughout the hospital stay.

Table 1 demonstrates the patient’s pertinent vital signs, laboratory results, and clinical information related to her itch symptoms (dialysis schedule, medication, and itch scale).

Initially, the nephrology team recommended initiating difelikefalin during hospitalization. The pharmacy department contacted the manufacturer; however, the medication could not be obtained because it is only approved and available for administration in the outpatient dialysis setting in the United States. A few days into her hospitalization, the patient developed significant complications, including acute decompensated heart failure, pneumonia, and an upper gastrointestinal bleed. Given these acute events, our multidisciplinary team determined that it was medically necessary to stabilize her before discharge. Only after stabilization would it have been appropriate and safe to initiate difelikefalin therapy through her outpatient dialysis center.

In this case, the decision-making process was guided by a multidisciplinary team that included nephrology, dermatology, pharmacy, and hospital medicine. The team jointly evaluated the patient’s acute complications, overall stability, and the feasibility of accessing difelikefalin in the inpatient setting. Given the inability to initiate difelikefalin during hospitalization and the patient’s severe ongoing pruritus, the team deliberated potential alternatives and agreed to trial low-dose naltrexone. Importantly, this decision was made with recognition that naltrexone use in uremic pruritus is off-label, and the rationale was to provide symptomatic relief in the context of medical instability while awaiting safe initiation of the licensed agent as an outpatient. All teams considered the importance of collaborative decision-making and the ethical considerations required when utilizing off-label pharmacotherapy in this complex clinical scenario.

Three days after oral naltrexone initiation 5 mg (1 mg/mL), the patient responded effectively. The itchiness level was assessed daily using visual analog scale (VAS) by staff dermatologists in the morning. Compared to pre-medication VAS score, the score reduced to an acceptable level reported by the patient by day 4–6 after naltrexone initiation. On day 4 of naltrexone use, the patient complained about the waning effect in the late afternoon. The nephrology team then increased the dose to 5 mg every 12 h. The itchiness level, assessed by both dermatology and nephrology teams using the VAS score, was at an acceptable level post dose increase.

The medication was compounded from a naltrexone oral tablet of 50 mg by the pharmacy department. Initially, the patient complained about the bitterness of the solution. The pharmacy subsequently mixed the compounded solution with Ora-Sweet to mask the bitterness. With regard to the side effects, two days after naltrexone initiation, the patient reported mild insomnia, which was relieved by melatonin 2 mg given at bedtime. The patient denied gastrointestinal symptoms (distress, dyspepsia or diarrhea). The patient also denied headache symptoms. Notably, the patient developed an upper gastrointestinal bleeding on day 4 of admission, which was 3 days before the initiation of naltrexone 5 mg. Subsequently, she was given pantoprazole 40 mg IV q 12 h for upper gastrointestinal bleeding and this was continued until discharge. Her itchiness was well controlled until her time of discharge on day 45 of hospitalization. Her hospital stay was complicated by acute decompensated heart failure, pneumonia, and chronic hypotension. She was discharged to a rehabilitation facility with an instruction to continue low-dose naltrexone given the improvement in the hospital.

## 3. Discussion

**Figure 1:**
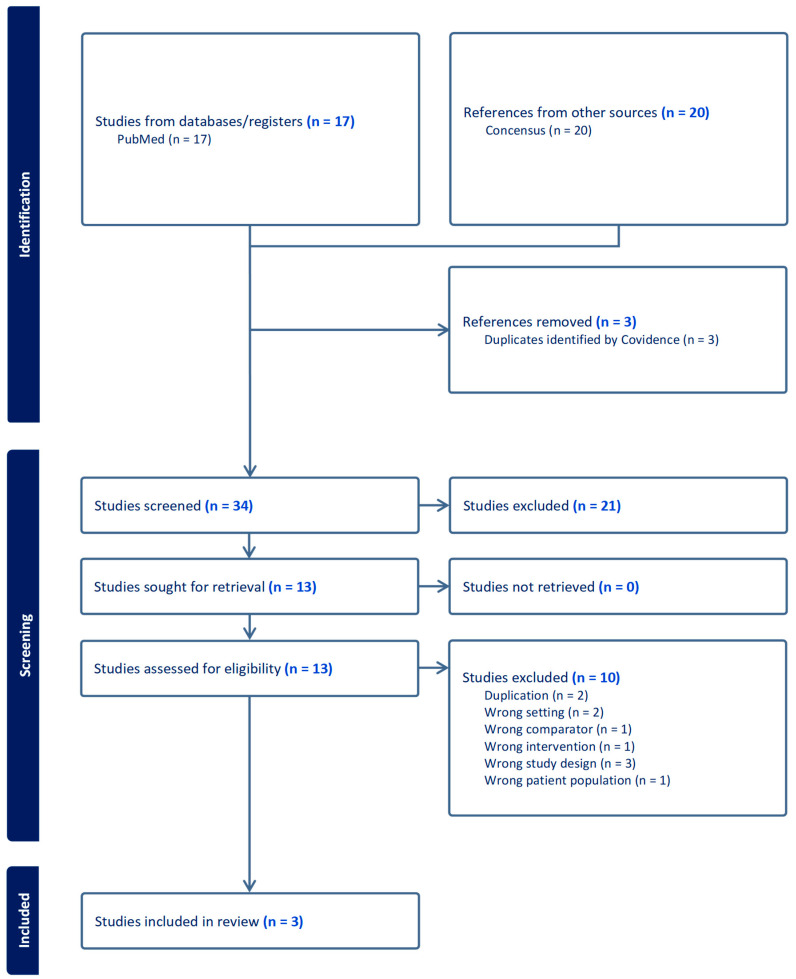
Preferred Reporting Items for Systematic Reviews and Meta-Analyses (PRISMA) Flowchart.

This case report presents a case in which the patient’s intractable itchiness, presumably due to uremic pruritus, was alleviated by low-dose naltrexone. There is severely limited information on the efficacy of low-dose naltrexone among patients with uremic pruritus. Based on our systematic initial literature search on PubMed, there was no published data on the use of low-dose naltrexone among uremic pruritus patients. Nevertheless, we found three studies discussing the effectiveness of naltrexone 50 mg in terms of the relief of itchiness among hemodialysis patients [12,13,14] (Table 2). We also enhanced our search by utilizing an artificial intelligence-powered search engine (Consensus-San Francisco, California) to ensure that we were not missing any key information. We were not able to identify additional studies. Figure 1 showed the Preferred Reporting Items for Systematic Reviews and Meta-Analyses (PRISMA) Flowchart for our studies included.

All three studies were classified as a high level of evidence (class 1) since they were all randomized controlled trials. However, no study showed a favorable outcome when naltrexone was used at 50 mg for the treatment of uremic pruritus among hemodialysis patients. This could be due to the fact that the high dose of naltrexone (50 mg) has different pharmacology and opioid receptor binding profiles. Opioid receptors are found ubiquitously in the human body, including the skin [15]. All three opioid receptors (μ, κ, and δ receptors) have expressions in keratinocytes, fibroblasts, and immune cells in the skin [15]. Importantly, μ opioid receptors have been implicated in stimulating itchiness, while κ opioid receptor activation has been shown to suppress itchiness [15,16]. Toll-like receptor 4 (TLR4) is increasingly recognized as a key player in the development of itchiness, particularly by contributing to the activation of spinal cord glial cells and signaling pathways that lead to an exaggerated itch sensation, especially in response to stimuli that would not normally trigger itchiness [17].

Low-dose naltrexone, given at a dose from 1 to 5 mg daily, functions differently than standard doses of 50–100 mg by only binding to μ, κ, and δ receptors for 4 to 6 h [18,19]. Low-dose naltrexone causes a short-term blockade of opioid receptors, which is hypothesized to lead to a rebound increase in endorphins and the upregulation of κ-opioid activity when the blockade wears off [18,19]. The increase in endogenous opioids and receptors could potentially modulate and decrease the itch signal by the activation of κ opioid receptors and the blockage of μ opioid receptors. Additionally, low-dose naltrexone also acts as an antagonist on Toll-like receptor 4 and could deactivate signaling pathways that lead to itch sensation [20]. Evidently, variations in dose utilization may result in different pharmacodynamic effects. This contrasts with the continuous blockade at 50 mg, which may not engage the same compensatory mechanisms and could explain why all three randomized studies were not able to provide successful outcomes since they utilized a higher dose of naltrexone of 50 mg.

In our case report, the patient is likely to have itchiness from uremic pruritus. The patient has normal serum total bilirubin and alkaline phosphatase, making the cholestatic pruritus is less likely. Normally, uremic pruritus patients often present with abnormalities of serum phosphate, calcium, and parathyroid hormone or have a history of missed dialysis, causing dialysis inadequacy. In this case, the patient presented with normal laboratory values. The patient’s BUN, Scr, PTH, and [Ca × P] product were within normal value ranges on admission, potentially suggesting the adequacy of dialysis (Kt/v was not available at the time of admission). In addition, medications from outpatient care were reviewed by staff dermatologists. Drug-induced pruritus was ruled out. Hence, the diagnosis of uremic pruritus was established by specialists from nephrology and dermatology care teams. After the patient was admitted, she was adequately dialyzed, as evident from her laboratory results (reduction in SCr and BUN). Gabapentin and antihistamines were started before the patient was admitted and were reportedly to be ineffective by the patient. The reduction in the VAS score was seen after the initiation of naltrexone. Therefore, we concluded that the improvement of the itch on day 9–10 could be a result of the initiation of low-dose naltrexone on day 7 of admission.

Side effects of naltrexone are dose-dependent; therefore, the use of a low dose might decrease the incidence of side effects. Common side effects include gastrointestinal distress, headache, insomnia, dizziness, and diarrhea. In our case, the patient only experienced mild insomnia, which was improved by melatonin. The fact that our patient did not report any gastrointestinal side effects could be due to the use of pantoprazole which was started on day 4 of admission for the treatment of upper gastrointestinal bleeding. Nonetheless, the use of low-dose naltrexone was proven to be safe and effective in this case.

While our case suggests that low-dose naltrexone may be a safe and potentially beneficial option for uremic pruritus, there is no commercially available dosage form for the “low dose” of naltrexone 1–5 mg. Typically, it has to be compounded by pharmacists. The information regarding the preparation and stability of naltrexone, as well as it compatibility with the admixture, was very limited. We gathered the available information and presented it in Table 3 [21,22,23]. We also consulted with a pharmaceutical scientist to ensure the accuracy of our recommendations. Our recommendations for compounding are as follows.

Compounding low-dose naltrexone: recommendations

- To prepare naltrexone oral suspension, in a mortar, crush 10 tablets of 50 mg naltrexone tablet with one 500 mg tablet of ascorbic acid, and mix to achieve a fine powder.
- Mix in a sodium benzoate 100 mg powder and levigate with 20 mL of 100% glycerin solution to form a paste.
- Gradually levigate sterile water into paste to form a liquid.
- Pour into a graduate cylinder.
- Rinse the mortar and add sterile water up to a total volume of 500 mL.
- If the sweetening agent or fruit juice was used, reduce the amount of sterile water and qs to 500 mL to make a 1 mg/mL solution.

In terms of the stability of naltrexone in the presence of sucrose or acidic juice, it is key that naltrexone is originally basic in structure and is prepared as the hydrochloride (HCl) salt. It is predicted that the medication will be stable in acidic medium given the fact that it is a HCl salt, and the structure contains a phenolic group. Therefore, it is not expected to see the release of protons at an acidic pH in a way that results in structural degradation. The preparation is also expected to be stable in sucrose solution.

## 4. Conclusions

Low-dose naltrexone may represent an attractive alternative treatment for refractory uremic pruritus, particularly in settings where difelikefalin is unavailable or its use is impractical. Its distinct pharmacological profile offers potential advantages over the traditional 50 mg dosing used in terms of opioid dependence. In this case, the patient tolerated therapy without significant adverse effects, suggesting that low-dose naltrexone could be considered as a viable option to alleviate severe CKD-associated pruritus. However, as this is a single case report, the findings should be interpreted with caution. Well-controlled studies are needed to determine the safety, efficacy, and appropriate role of low-dose naltrexone in uremic pruritus.

## Figures and Tables

**Table 1:** Patient’s clinical parameters, dialysis schedule, medications related to pruritus, and itch severity scores during hospitalization.

Parameter	Day 1	Day 2	Day 3	Day 4	Day 5	Day 6	Day 7	Day 8	Day 9	Day 10	Day 13	Day 14
**Vitals**												
SBP (mmHg)	140	114	103	105	127	122	129	143	134	118	123	111
DBP (mmHg)	90	99	52	61	56	56	59	74	68	64	58	43
HR (BPM)	150	160	72	64	77	90	89	105	93	110	74	72
Temperature (F)	98.1	98.4	98.0	97.5	98.2	98.9	96.5	98.4	97.0	98.9	97.8	97.7
QTC (ms)						474	485	489	494	491		509
**Chemistry**												
Na (mEq/L)	141	140	139	128	131	131	130	127	134	137	140	140
K (mEq/L)	4.7	4.3	4.1	4.1	4.1	4.1	4.0	3.9	4.2	4.3	3.7	4.0
Glucose (mg/dL)	71	119	174	174	169	154	143	154	160	129	154	139
BUN (mg/dL)	22	33	21	52	47	38	34	46	31	40	34	41
Scr (mg/dL)	3.69	5.08	3.57	4.88	5.24	3.66	3.76	4.24	3.76	4.75	4.58	5.53
Ca (mg/dL)	8.4	8.6	8.4	9.1	7.2	7.3	7.2	7.3	7.4	7.3	7.5	7.8
Phos (mg/dL)	3.5	3.5	2.6	3.1	3.0	2.1	2.7	4.8	5.3	4.4	4.2	6.0
Corrected Ca (mg/dL)	8.9	9.1	8.9	9.6	8.5	8.6	8.5	8.6	8.7	8.6	8.8	9.1
[Ca × P] product	31	32	23	29	25	18	23	41	46	38	37	54
PTH (pg/mL)									339			
Albumin (g/dL)	3.28				2.28							2.30
25(OH)D (ng/mL)									23.4			
Total bilirubin (mg/dL)	0.6				0.9							
Alkaline phosphatase (IU/L)	122				100							
**Medication administration related to pruritus**												
Gabapentin 100 mg PO q 24 h.	x	x	x	x								
Gabapentin 100 mg PO q 8 h.					x	x	x	x	x			
Diphenhydramine 25 mg IV PRN	x											
Hydroxyzine 50 mg PO q 8 PRN	x	x		x	x	x	x					
Naltrexone 5 mg (1 mg/mL) PO q 24 h.							x	x	x			
Naltrexone 5 mg (1 mg/mL) PO q 12 h.									x	x	x	x
**Hemodialysis treatment**												
Day of treatment		x	x		x			x		x		x
**Patient reported itchiness level on visual analog scale (VAS)**												
VAS (10)	10	10	10	10	10	10	10	9	6	4	3	3

Abbreviations: mg: milligram, mL: milliliter, VAS: visual analog scale, PO: by month, mEq: milliequivalent, L: liter, dL: deciliter, BPM: beat per minute, pg: picogram, IU: international unit, PRN: as needed. ng: nanogram, F: Fahrenheit.

**Table 2:** Key studies and outcomes on the use of naltrexone among hemodialysis patients.

Type of Study	Population	Intervention	Comparator	Outcome	Reference
Randomized, double-blinded, placebo-controlled crossover trial	15 chronic hemodialysis patients	Naltrexone 50 mg q 24 h given by mouth	Placebo	Naltrexone was minimally efficacious. Naltrexone 50 mg modulates histamine release, not by an anti-opioid effect.	Peer, 1996 [12]
Randomized, double-blinded, placebo-controlled crossover trial	23 patients suffered from uremic pruritus	Naltrexone 50 mg q 24 h given by mouth	Placebo	The difference between naltrexone vs. placebo for the relief of pruritus was not statistically significant	Pauli-Magnus, 1999 [13]
Randomized study	52 patients suffered from uremic pruritus	Naltrexone 50 mg q 24 h given by mouth	Loratadine 10 mg given orally	There was no significant difference in the mean VAS scores after treatment.	Legroux-Crespel, 2003 [14]

**Table 3:** Information on stability and compatibility of the compounded low-dose naltrexone.

Dosage	Diluent	Concentration	Flavoring/Sweetening	Stability of Oral Liquids Prepared from Powder	Reference
Naltrexone oral tablets (50 mg)	Sterile water Ascorbic acid 0.5% Sodium benzoate 0.1% Glycerin 20%	1 mg/mL	None	Stored in the dark, stable for 90 days (room temperature or refrigerated)	Fawcett, 1997 [22]
		5 mg/mL	None	Stored in the dark, stable for 60 days (room temperature or refrigerated)	Fawcett, 1997 [22]
	Drinking water	1 mg/mL	Juice	3 months when refrigerated	Bronfenbrener, 2021 [23]
Naltrexone oral tablets (50 mg)	N/A	N/A	Natural sweetener (Monk fruit)	180 days in the refrigerator and at room temperature	Pramar, 2019 [21]

## Data Availability

The data presented in this study are available on request from the corresponding author.
